# Editorial: Transitioning through menopause: contraceptive choices and sexual well-being

**DOI:** 10.3389/frph.2026.1916638

**Published:** 2026-07-16

**Authors:** Hannah Sebitloane, Rosie Mngqibisa

**Affiliations:** 1 Department of Obstetrics and Gynaecology, Univeristy of Kwazulu Natal, Durban, South Africa; 2 Enhancing Care Foundation, Durban, South Africa

**Keywords:** intergrated approach, menopause, menopause quality of life, menopause transition, sexual health

The menopausal transition (MT) is a period of fluctuating hormones, changing fertility, altered sexual function, and evolving contraceptive needs. MT is a pivotal phase in a woman's life characterized by significant physiological, psychological, and social transformations. Despite its universality, the nuances of how it affects various aspects of women's health and well-being, particularly contraceptive use and sexual well-being, remain poorly understood. MT period starts years before permanent cessation of menses, affects many systems and increases the risk of cardiometabolic health–related changes due to alterations in endogenous sex hormones, changes in body fat distribution, lipid metabolism, as well as structural and functional measures of vascular health (1). Commonly reported symptoms include vasomotor symptoms (hot flushes/night sweats), genitourinary syndrome (GSM) symptoms such as vaginal dryness and dyspareunia, sleep and mood dysfunction and irregular menses. Many of these symptoms affect sexual well-being and quality of life for women.

During MT, women go through a complex phase in which fertility declines but does not disappear. As ovarian reserves wane, ovulation is unpredictable and can occur intermittently. Consequently, clinicians must balance prescription of contraceptives with symptom management through individualized hormonal and nonhormonal strategies. The aim of this Research Topic of articles is to seek to answer critical questions about the best contraceptive practices during this period and how menopausal changes affect sexual health. Additionally, it aims to investigate the broader cultural and social narratives surrounding menopause and how they influence women's experiences and health care approaches.

Since menstrual irregularity does not equal infertility, pregnancy remains possible until menopause is confirmed, and thus contraceptive consideration is essential. This can affect sexual well-being because fear of unintended pregnancy may reduce sexual spontaneity, increase anxiety and affect intimacy, thus altering relationship dynamics. There needs to be a structured, intentional discourse between the clinicians and women in MT about their contraceptive concerns and sexual well-being. The study by Li et al. demonstrated uncertainties about contraception during this period by studying the best time and how to safely remove an intrauterine contraceptive device (IUCD) in postmenopausal women where the device may have been left *in situ* for longer than is necessary, due to lack of “safe space” platforms for such an interaction. This study highlights failures and reasons for failure to remove the IUCD in the cohort enrolled. These factors include uterine atrophy, hormonal changes, device aging, and embedding. The study highlights an important gap as IUCD is one of the commonly used forms of contraception.

While we did not receive articles directly addressing the broader perspective of contraceptive options in this very important phase of a woman's life the other topics addressed in the submitted articles are nonetheless very relevant to sexual wellbeing of perimenopausal women. It is important to note that because of the interplay between hormonal changes and sexual wellbeing, the choice of birth control can either exacerbate or alleviate sexual dysfunction during MT. Some older combined oral contraceptives with high levels of synthetic estrogens may suppress free testosterone concentrations through elevations in sex hormone-binding globulin, potentially contributing to reduced libido and diminished responsiveness to erotic stimuli (2). Current literature recommends prioritizing newer contraceptive formulations containing natural estrogens, such as estradiol, as these appear to exert less hepatic stimulation and may minimize SHBG-related reductions in androgen bioavailability and sexual function (2).

Sexual well-being during MT is multidimensional and requires an integrated biopsychosocial and health-systems approach. Sexual health is not only a hormonal or gynaecological issue, but also a matter of public policy, clinical risk management, technological innovation, interpersonal relationships, and quality-of-life outcomes. Sexual well-being during MT and postmenopausal phases is shaped by the interaction of biological aging and hormonal changes, and symptom management strategies. Underscoring all these factors is the need to balance emotional, relational reproductive health needs within the broader public health systems. Therefore, effective menopausal care requires integrated, woman-centered, multidisciplinary approaches which include institutional and community support that address both physical and psychosocial dimensions of health. This positions menopause as a public health gender equity issue, and a policy priority. The article by Selva identiﬁes governmental proposals and speciﬁc actions to improve support for the menopausal transition and post-menopause and lay the groundwork for developing public policies and laws that respond to the needs of women, improving their well-being and promoting gender equity in public health. This work recognizes that perimenopausal sexual health cannot be optimized without supportive healthcare systems and public policy structures. Further work by Faila and Lenz, in this collection demonstrates that partnership improves outcomes in women receiving HRT citing that “a stable partnership may facilitate the integration of psychobiological processes of hormonal changes and contribute to improved subjective adaptation to menopause”. Both these articles create the structural foundation upon which individualized interventions and clinical management rest and support the notion that sexual well-being is implicitly connected through improved healthcare access, menopause education, psychosocial support, workplace inclusion, and reduction of stigma. Menopause experiences are socially embedded, and supportive relationships can significantly shape therapeutic outcomes and subjective well-being. Symptoms of menopause are often treated with HRT, however, there are psychosocial and relational dimensions to wellbeing which are often neglected. This study shows that partnership status influences symptom perception, adaptation to menopause, and quality-of-life outcomes during HRT (Faila and Lenz). This reinforces the idea that sexual well-being is relational and emotional, not merely biological. Partnership may influence emotional security, intimacy, body image, sexual communication, and treatment responsiveness. It strengthens the biopsychosocial model of menopause. Sexual well-being should be seen s as an indicator of healthy aging, bodily autonomy, relational satisfaction, and psychosocial adaptation. Sexual health is therefore not peripheral, but central to menopause care.

When delving into treatment during MT, it is important to note that not all menopausal symptoms can be alleviated through hormones or even newer non-hormonal options such as selective neurokinin 3 receptor antagonists. There remains an urgent need to develop new options to deliver effective treatment for women experiencing these symptoms. Options such as CO₂ laser, a non-hormonal intervention for GSM symptoms and vulvovaginal tissue changes are needed. The findings from the study by Hatta et al. suggest that fractional CO₂ laser therapy was associated with improvements in GSM symptoms, particularly dyspareunia, as well as increased labia majora thickness and high levels of patient satisfaction. This therapy is thought to relieve painful intercourse, restore sexual function and genital comfort, and quality of life. Such innovative and individualized therapies can improve sexual wellbeing and reduces discomfort in postmenopausal women. Importantly, there's a growing movement toward personalized medicine, non-hormonal alternatives, and quality-of-life-centered menopause care.

Collectively, these studies demonstrate that sexual well-being during perimenopause is shaped by interconnected biological, psychosocial, reproductive, and structural factors. Effective menopausal care therefore requires integrated models that combine public health policy, individualized clinical management, innovative therapeutic interventions, and attention to relational and emotional well-being. Together, these approaches support a broader reconceptualization of perimenopause from a narrowly biomedical event to a multidimensional life transition that significantly influences women's quality of life and sexual health, and thus require a high level of intentionality by clinicians treating women in MT. This includes understanding women's subjective experiences of MT and what it means for their sexuality and relationships, review of their medical comorbidities and medications with sexual side effects and adjust when needed. Additionally, it is important to screen for GSM and proactively treat it. The findings are not exhaustive but only scratch the surface of what clinicians should be thinking about when discussing sexual health with their MT patients.
